# Supplementary material for: Mortality among People with Epilepsy: A Retrospective Nationwide Analysis from 2016 to 2019
Source: Int J Environ Res Public Health. 2021 Oct 7;18(19):10512. doi: 10.3390/ijerph181910512 (PMC8508525; doi:10.3390/ijerph181910512)
Supplement: Supplementary file 1 [file ijerph-1392786-supplementary.pdf › ijerph-1392786-supplementary.pdf]

Supplementary Table S1. The frequency of different causes of death.

	Year (number of deaths, % of all deaths that year)																							
	**2016**						**2017**						**2018**						**2019**					
	**All**		**Male**		**Female**		**All**		**Male**		**Female**		**All**		**Male**		**Female**		**All**		**Male**		**Female**	
	**n**	**%**	**n**	**%**	**n**	**%**	**n**	**%**	**n**	**%**	**n**	**%**	**n**	**%**	**n**	**%**	**n**	**%**	**n**	**%**	**n**	**%**	**n**	**%**
**Causes of death**																								
**External causes of death**	186	10.9	160	13.9	26	4.7	161	10.4	132	13.5	29	5	145	9.6	117	12	28	5.3	135	8.5	114	11.5	21	3.5
Falls	36	2.1	27	2.3	9	1.6	37	2.4	28	2.9	9	1.6	35	2.3	28	2.9	7	1.3	35	2.2	27	2.7	8	1.3
Transport accidents	10	0.6	9	0.8	1	0.2	7	0.5	6	0.6	1	0.2	9	0.6	9	0.9	0	0	4	0.3	3	0.3	1	0.2
Drowning	10	0.6	7	0.6	3	0.5	8	0.5	7	0.7	1	0.2	5	0.3	2	0.2	3	0.6	8	0.5	8	0.8	0	0
Poisoning	29	1.7	26	2.3	3	0.5	17	1.1	14	1.4	3	0.5	19	1.3	17	1.7	2	0.4	16	1	15	1.5	1	0.2
Suicide	34	2	34	3	0	0	32	2.1	28	2.9	4	0.7	19	1.3	18	1.8	1	0.2	17	1.1	16	1.6	1	0.2
Assault	4	0.2	3	0.3	1	0.2	2	0.1	1	0.1	1	0.2	5	0.3	5	0.5	0	0	4	0.3	4	0.4	0	0
Event of undetermined intent	20	1.2	17	1.5	3	0.5	17	1.1	16	1.6	1	0.2	19	1.3	16	1.6	3	0.6	20	1.3	15	1.5	5	0.8
**Infectious diseases**	47	2.8	34	3	13	2.4	30	1.9	21	2.2	9	1.6	25	1.7	18	1.8	7	1.3	29	1.8	17	1.7	12	2
Sepsis	30	1.8	22	1.9	8	1.5	9	0.6	4	0.4	5	0.9	12	0.8	6	0.6	6	1.1	15	0.9	7	0.7	8	1.3
**Cancer**	283	16.7	189	16.4	94	17.1	268	17.2	166	17	102	17.6	321	21.3	202	20.7	119	22.4	300	18.9	199	20.1	101	16.9
Cancer of trachea, bronchus, and lung	53	3.1	47	4.1	6	1.1	45	2.9	36	3.7	9	1.6	55	3.6	44	4.5	11	2.1	53	3.3	44	4.4	9	1.5
Breast cancer	12	0.7	0	0	12	2.2	13	0.8	0	0	13	2.2	19	1.3	0	0	19	3.6	14	0.9	0	0	14	2.3
Cervical cancer	6	0.4	0	0	6	1.1	6	0.4	0	0	6	1	3	0.2	0	0	3	0.6	6	0.4	0	0	6	1
Prostate cancer	17	1	17	1.5	0	0	7	0.5	7	0.7	0	0	11	0.7	11	1.1	0	0	10	0.6	10	1	0	0
Brain cancer	76	4.5	46	4	30	5.5	68	4.4	36	3.7	32	5.5	83	5.5	39	4	44	8.3	68	4.3	40	4	28	4.7
**Endocrine, nutritional and metabolic diseases**	8	0.5	7	0.6	1	0.2	11	0.7	7	0.7	4	0.7	15	1	7	0.7	8	1.5	17	1.1	13	1.3	4	0.7
**Neurological and psychiatric diseases**	107	6.3	68	5.9	39	7.1	127	8.2	78	8	49	8.5	110	7.3	66	6.7	44	8.3	133	8.4	90	9.1	43	7.2
Epilepsy	36	2.1	25	2.2	11	2	45	2.9	26	2.7	19	3.3	39	2.6	27	2.8	12	2.3	43	2.7	31	3.1	12	2
Status epilepticus	8	0.5	3	0.3	5	0.9	9	0.6	8	0.8	1	0.2	9	0.6	8	0.8	1	0.2	5	0.3	4	0.4	1	0.2
Dementia (including Alzheimer's disease)	14	0.8	5	0.4	9	1.6	17	1.1	9	0.9	8	1.4	11	0.7	8	0.8	3	0.6	17	1.1	10	1	7	1.2
**Cardiovascular diseases**	838	49.3	505	43.9	333	60.7	757	48.7	440	45.1	317	54.7	677	44.8	397	40.6	280	52.6	749	47.1	407	41	342	57.3
Ischemic heart disease	437	25.7	248	21.6	189	34.4	407	26.2	246	25.2	161	27.8	334	22.1	193	19.7	141	26.5	388	24.4	200	20.2	188	31.5
Stroke	160	9.4	94	8.2	66	12	152	9.8	84	8.6	68	11.7	125	8.3	71	7.3	54	10.2	133	8.4	74	7.5	59	9.9
**Diseases of the respiratory system**	46	2.7	42	3.7	4	0.7	37	2.4	25	2.6	12	2.1	51	3.4	45	4.6	6	1.1	50	3.1	38	3.8	12	2
Pneumonia	24	1.4	23	2	1	0.2	18	1.2	11	1.1	7	1.2	27	1.8	24	2.5	3	0.6	27	1.7	22	2.2	5	0.8
Chronic respiratory diseases	16	0.9	13	1.1	3	0.5	13	0.8	9	0.9	4	0.7	19	1.3	19	1.9	0	0	13	0.8	9	0.9	4	0.7
**Diseases of the digestive system**	123	7.2	101	8.8	22	4	103	6.6	73	7.5	30	5.2	106	7	84	8.6	22	4.1	103	6.5	71	7.2	32	5.4
Liver diseases	80	4.7	70	6.1	10	1.8	59	3.8	43	4.4	16	2.8	72	4.8	57	5.8	15	2.8	63	4	48	4.8	15	2.5
**Diseases of the genitourinary system**	16	0.9	12	1	4	0.7	16	1	12	1.2	4	0.7	12	0.8	11	1.1	1	0.2	16	1	9	0.9	7	1.2
**Unknown and unspecified causes**	14	0.8	12	1	2	0.4	14	0.9	12	1.2	2	0.3	21	1.4	19	1.9	2	0.4	28	1.8	21	2.1	7	1.2
**Diseases of the skin and subcutaneous tissue**	2	0.1	2	0.2	0	0	0	0	0	0	0	0	2	0.1	1	0.1	1	0.2	0	0	0	0	0	0
**Diseases of the musculoskeletal system and connective tissue**	3	0.2	2	0.2	1	0.2	4	0.3	3	0.3	1	0.2	4	0.3	3	0.3	1	0.2	3	0.2	1	0.1	2	0.3
**Congenital malformations and chromosomal abnormalities**	7	0.4	3	0.3	4	0.7	8	0.5	2	0.2	6	1	6	0.4	3	0.3	3	0.6	7	0.4	5	0.5	2	0.3
**Other neoplasms and diseases of blood and blood-forming organs**	17	1	11	1	6	1.1	17	1.1	4	0.4	13	2.2	15	1	5	0.5	10	1.9	19	1.2	7	0.7	12	2
**Symptoms and ill-defined conditions (other than unknown causes)**	1	0.1	1	0.1	0	0	0	0	0	0	0	0	0	0	0	0	0	0	0	0	0	0	0	0
**Diseases of the eye and ear**	1	0.1	1	0.1	0	0	0	0	0	0	0	0	0	0	0	0	0	0	0	0	0	0	0	0
**Pregnancy, childbirth and puerperium**	0	0	0	0	0	0	1	0.1	0	0	1	0.2	0	0	0	0	0	0	0	0	0	0	0	0
